# Telesimulation Use in Emergency Medicine Residency Programs: National Survey of Residency Simulation Leaders

**DOI:** 10.5811/westjem.24863

**Published:** 2024-10-22

**Authors:** Max Berger, Jack Buckanavage, Jaime Jordan, Steven Lai, Linda Regan

**Affiliations:** * University of California Los Angeles, David Geffen School of Medicine, Department of Emergency Medicine, Los Angeles, California; † Mount Sinai School of Medicine, Department of Emergency Medicine, New York, New York; ‡ Johns Hopkins University, Department of Emergency Medicine, Baltimore, Maryland

## Abstract

**Introduction:**

Coronavirus 2019 (COVID-19) accelerated the need for virtual learning including telesimulation. Many emergency medicine (EM) programs halted in-person simulation and trialed telesimulation, but specifics on its utilization and plans for future use are unknown. Telesimulation has been defined as “a process by which telecommunication and simulation resources are utilized to provide education, training, and/or assessment to learners at an off-site location.” Our objective in this study was to describe the patterns of telesimulation usage in EM residency programs during COVID-19-induced learning restrictions as well as its anticipated future utility.

**Methods:**

We identified EM simulation leaders via the EMRA Match website, institutional websites, or personal contact with residency coordinators and directors, and invited them to participate by email. Participants completed a confidential, web-based survey consisting of multiple-choice items and one free-response question, developed by our study team with consideration of survey research best practices and Messick’s validity framework. We collected data between January–February 2022. We calculated descriptive statistics for multiple-choice items and examined the free-response answers for common themes.

**Results:**

We obtained contact information for simulation leaders at 139 EM residency programs. Survey response rate was 65% (91/139). During in-person restrictions, 62% (56/91) of programs used telesimulation. Assuming all restrictions lifted, 38% (34/90) of respondents planned to continue to use telesimulation, compared to 9% (8/91) using telesimulation before COVID-19. Most respondents planned to use telesimulation for medical knowledge (26/34, 76%) and communication/teamwork-focused cases (23/34, 68%). In response to the free-response question regarding experience with and plans for use, we identified three major themes: 1) telesimulation is a valuable alternative to in-person learning; 2) telesimulation is an option for learners unable to participate in person; and 3) telesimulation is challenging for procedural education.

**Conclusion:**

Despite the relatively limited use of telesimulation in EM residencies prior to COVID-19, an increased number of programs have plans to continue incorporating telesimulation into their curricula. This plan for continued use opens opportunities for further innovation and scholarship within simulation education.

Population Health Research Capsule What do we already know about this issue?

*COVID-19 accelerated the need for virtual learning including telesimulation.*

What was the research question?

*To what extend was telesimulation used by EM residencies during COVID-19, and what is its anticipated utility moving forward?*

What was the major finding of the study?

*Only 9% (8/91) of programs used telesimulation before COVID-19. During COVID restrictions, 62% (56/91) of programs used it, while after limitations were lifted, 38% (34/90) planned to continue telesimulation.*

How does this improve population health?

*As an adjunct to traditional in-person simulation curriculum, telesimulation is a viable option to improve medical knowledge and communication-based competencies.*

## INTRODUCTION

Restrictions imposed on in-person education during the coronavirus 2019 (COVID-19) pandemic accelerated the need for virtual learning, including telesimulation.^1^ ^,^ ^2^ Telesimulation has been defined as “a process by which telecommunication and simulation resources are utilized to provide education, training, and/or assessment to learners at an off-site location.”^3^ Initial applications were in lower resource settings such as developing countries where learners did not have access to simulation centers or instructors.^4^ ^,^ ^5^ Within telesimulation, different modalities have been described that vary in fidelity as well as location of the learners and instructors relative to each other and the simulation center.^6^ ^–^ ^8^

Several published articles since early 2020 have described different institutions’ approaches to telesimulation since the pandemic.^1^ ^–^ ^2^ ^,^ ^9^ ^–^ ^13^ Common themes include the need to modify learning objectives to virtual environments and to select the appropriate modality of telesimulation based on institutional needs and resources.^9^ ^–^ ^12^ Different modalities of telesimulation have been described, including the following: 1) learners virtually observing and debriefing a live simulation^7^; 2) learners present with a manikin while instructors facilitate from a separate location^6^; 3) instructors present with a manikin while learners remotely participate^7^; and 4) completely remote option where learners and instructors both participate remotely from separate locations.^10^ ^,^ ^11^

Limited data comparing telesimulation vs traditional simulation suggests that learner satisfaction with telesimulation or hybrid virtual and in-person simulation is similar, although this was not found in all studies.^7^ ^,^ ^13^ ^,^ ^14^ A scoping review from 2021 highlighted the mixed data on student perception of telesimulation, with some of the included studies indicating remote facilitation of simulation being perceived as equally or more effective than live facilitation, while others found remote facilitation to be inferior.^14^ Facilitator perception of telesimulation has not been well studied. Limited learning outcome data has suggested similar improvements between in-person simulation and telesimulation.^8^ ^,^ ^14^

Our objective in this study was to describe the patterns of telesimulation usage in emergency medicine (EM) residency programs during COVID-19-induced, in-person learning restrictions as well as its anticipated utility moving forward. This information is crucial to understanding the value of telesimulation and its utility in medical education.

## METHODS

### Study Design, Setting, and Population

We conducted a cross-sectional survey study of faculty in charge of simulation for EM residency programs in the United States. We collected data from January–February 2022. After identifying EM residency programs and their websites from the EMRA Match database,^15^ we searched each website for contact information for the director of simulation education. If there was no director designated, we emailed each residency’s program coordinator and/or director asking for contact information for the faculty in charge of residency simulation. Each program was allowed only one designated participant. This study was given exempt status by the University of California, Los Angeles Institutional Review Board (IRB#21-001336) and the Johns Hopkins University Homewood IRB (HIRB00013694).

### Survey Development and Dissemination

Given the lack of any previously created survey applicable to this construct, the primary author (MB) developed a web-based survey tool with consideration of survey research best practices and Messick’s validity framework.^16^ ^–^ ^19^ For content validity evidence, we first performed a literature review, and the author group of expert simulation educators and medical education researchers reviewed the survey for clarity and relevance to the construct. We defined telesimulation as including any simulation activity where “telecommunication and simulation resources are utilized to provide education, training, and/or assessment to learners at an off-site location.”^3^ We piloted the survey with a group of simulation educators who were not included in the target sample to gather response process validity evidence. After piloting, we revised the survey for clarity. The final survey included multiple-choice items and one free-text response item (Appendix 1).

We invited participants by email and sent two targeted, follow-up invitations to non-responders at bi-weekly intervals. We administered the survey via Qualtrics (Qualtrics, LLC, Provo, UT). No individual identifying information was collected. To maximize response rate and minimize guessing, we did not require participants to complete all survey items. Participants were not compensated for participating in the study.

### Data Analysis

We calculated and reported descriptive statistics for items with discrete answers. We conducted calculations using Qualtrics and Microsoft Excel for Mac (Microsoft Corp, Redmond, WA). We examined the answers to the free-text responses to identify common themes that would broaden the reader’s understanding of the data. Successive wave analysis was performed to assess the extent of possible nonresponse bias.^20^ We examined whether use of telesimulation during the pandemic, planned future use of telesimulation after in-person restrictions, and respondent program format (postgraduate years [ PGY] 1–3 vs 1–4) differed by wave. Bivariate chi-square tests for each variable of interest by wave were performed using Microsoft Excel for Mac, and *P*-values less than 0.05 were considered statistically significant. We used the consensus-based checklist for reporting of survey studies (CROSS) as reporting guidelines (Appendix 2).^23^

## RESULTS

Of 139 simulation leaders we identified, 91 (65%) completed the survey with 87 (63%) completing all items. We report demographic data for survey respondents in Table 1, while respondents’ experience and perceptions of telesimulation are shown in Table 2. Prior to the COVID-19 pandemic, 9% (8/91) of survey respondents were using telesimulation in their curricula. There was a wide variety of prior experiences with telesimulation, with the most common being that they had heard of telesimulation but never been involved (44%). Ninety-two percent (84/91) of respondents reported that their institution prohibited in-person learning activities at some point during the COVID-19 pandemic. During in-person learning restrictions, 62% (56/91) used telesimulation in some form.

**Table 1.** Survey respondent demographics.

	n (%)
Format of respondent’s current residency program	
PGY 1–3	62/89 (70%)
PGY 1–4	27/89 (30%)
Size of respondent’s current residency program (total number of residents in all years)	
≤20 residents	11/90 (12%)
21–40 residents	38/90 (43%)
41–60 residents	31/90 (34%)
≥60 residents	10/90 (11%)
Respondent’s current residency program primary institution setting	
University-based	58/90 (64%)
Community-based	28/90 (31%)
County-based	13/90 (14%)
Prior simulation training of survey respondent	
Fellowship training in simulation	31/90 (34%)
Non-fellowship training in simulation	48/90 (53%)
No formal training in simulation	17/90 (19%)
Respondent years since residency graduation	
≤5	22/90 (24%)
6–10	33/90 (37%)
11–15	16/90 (18%)
16–20	8/90 (9%)
≥21	11/90 (12%)

*PGY*, postgraduate year.

**Table 2.** Key survey results.

	n (%)
EM residency program use of telesimulation	
Prior to COVID-19 pandemic	8/91 (9%)
During in-person learning restrictions	56/91 (62%)
Planned use after in-person restrictions lifted	34/90 (38%)
During any point in the COVID-19 pandemic, did your institution prohibit in-person learning activities?	
Yes	84/91 (92%)
No	7/91 (8%)
Experience with telesimulation prior to COVID-19	
Had never heard of telesimulation	17/91 (19%)
Heard of telesimulation but never involved	40/91 (44%)
Attended a presentation	20/91 (22%)
Participated as an instructor	19/91 (21%)
Participated as a learner	6/91 (7%)
Conducted a research project	5/91 (5%)
Read a paper about telesimulation	16/91 (18%)
Formats of telesimulation used during COVID-19 restrictions	
Completely virtual; utilizing real-time patient monitor and/or manikin	21/90 (23%)
Completely virtual; oral boards style cases	31/90 (35%)
Hybrid; instructor, learners and/or sim tech in sim center while others remote	31/90 (35%)
What simulation activities were best suited for telesimulation?	
Medical knowledge focused cases	65/90 (72%)
Communication/teamwork focused cases	42/90 (47%)
Procedure focused cases	5/90 (6%)
Dedicated procedure training	2/90 (2%)
Procedure training on homemade models	10/90 (11%)
What simulation activities were not well suited for telesimulation?	
Medical knowledge focused cases	0/87 (0%)
Communication/teamwork focused cases	18/87 (21%)
Procedure focused cases	52/87 (60%)
Dedicated procedure training	54/87 (62%)
Procedure training on homemade models	23/87 (26%)
Percent of future simulation curriculum involving telesimulation	
0% of the curriculum	56/90 (62%)
1–25% of the curriculum	30/90 (33%)
26–50% of the curriculum	3/90 (3%)
51–75% of the curriculum	1/90 (1%)
76–100% of the curriculum	0/90 (0%)
Types of future simulation activities for those who plan to continue using telesimulation	
Medical knowledge-focused cases	26/34 (76%)
Communication/teamwork-focused cases	23/34 (68%)
Procedure focused cases	7/34 (21%)
Dedicated procedural training	5/34 (15%)
Procedure training on homemade models	5/34 (15%)

*EM*, emergency medicine.

When survey respondents were asked about what format(s) of telesimulation were used, 11% (10/90) stated that they only used a completely virtual oral-boards style format, while the rest of those who used telesimulation reported using a hybrid or virtual format involving a patient monitor and/or manikin. The largest percentage of survey respondents felt that medical knowledge and communication/teamwork-focused cases were best suited for telesimulation (72% and 47% respectively), while most felt that procedure training was not well suited for telesimulation (62%). Thirty-eight percent (34/90) of respondents stated they planned to use telesimulation in some form in their curriculum moving forward, mostly for medical knowledge and communication/teamwork-focused cases (76% and 68%, respectively).

We received 14 free-text responses, and identified three major themes, described below with exemplar quotes.

1. Telesimulation is a valuable alternative to in-person learning:“It has been the ‘better than nothing’ option but accepted by learners when other options are not feasible.”“It has exceeded expectations in how helpful it has been.”
2. Telesimulation is an option for learners unable to participate in person:“We found that it’s a great option for residents with families or who have other extenuating circum-stances why they can’t participate in person, ie, breastfeeding moms, new parents, elder care, etc. Many of our residents who are between nights or between mid-shifts will log on and participate.”
3. Telesimulation is challenging for procedural education:“Difficult to learn muscle memory for high acuity, low occurrence skills.”“Procedural training was the most difficult to simulate via telesim.”

For the wave analysis, the study included 91 respondents, including 42 in wave 1 (46%), 21 in wave 2 (23%), and 28 in wave 3 (31%). Results of the examined survey questions did not statistically differ by wave with all *P*-values > 0.05. (See Supplemental Table.)

## DISCUSSION

Despite relatively low use of telesimulation within EM programs prior to the COVID-19 pandemic, we found that many EM residency programs (62%) quickly adapted to in-person learning restrictions by using telesimulation. While not all programs that trialed telesimulation plan to continue its use, 38% of respondent programs do plan to continue to use telesimulation, compared to 9% of programs using telesimulation prior to COVID-19. This represents a large increase in the overall usage of telesimulation within EM residencies. Our study also sheds light on how telesimulation can benefit EM programs. Being able to increase learner participation to include residents with family obligations or between night shifts could allow for increased return on investment for simulation resources and faculty time. Most respondents who plan to continue to use telesimulation reported that they will use it as a small percentage of their overall simulation curriculum, which highlights that telesimulation is not replacing in-person simulation but augmenting the traditional curriculum. This could be in a hybrid format that allows for increased participation, or as part of separate telesimulation days that could reduce the travel burdens on learners and instructors.

There was large variation in how programs conducted telesimulation during in-person restrictions. This is in line with prior literature and likely reflects individual program needs, preferences, and available resources.^1^ ^,^ ^2^ ^,^ ^8^ ^–^ ^11^ ^,^ ^13^ ^,^ ^22^ Most described telesimulation as best suited for medical knowledge and communication/teamwork-focused cases, rather than for procedure teaching. This is interesting given that early descriptions of telesimulation in the literature mostly involved procedural teaching.^5^ ^,^ ^6^ One possible explanation for this discrepancy is that those early studies involved duplicate simulators at remote locations, an expense that is likely not practical, or necessary, for a residency program given the ability to host procedure training as part of the in-person curriculum. While it is apparent that there are increased plans for the use of telesimulation compared to the pre-pandemic era, not all residency programs who used telesimulation during times of in-person restrictions are planning to continue to do so. The reasons for this are unknown but may relate to telesimulation resource availability or limited outcome data on its utility.

Based on our results, we believe that telesimulation can continue to be a valuable addition to the traditional in-person simulation curriculum, particularly in allowing for increased participation of learners and instructors, reducing resource costs such as simulation center and staff time, and allowing for a viable option to practice medical knowledge and communication-based competencies. Now that telesimulation has been established as an instructional strategy that will continue to be part of many EM residency curricula, it opens opportunities for future innovation and scholarship within simulation-based medical education. Additional investigation could compare different modalities of telesimulation on objective learning outcomes.^23^ It would also be interesting to explore the role of virtual and augmented reality within telesimulation.^24^ ^,^ ^25^

## LIMITATIONS

Despite multiple attempts, we were not able to obtain contact information for a simulation leader from all EM programs. However, the breakdown of PGY 1–3 vs PGY 1–4 programs of survey respondents (70% PGY 1–3 vs 30% PGY 1–4), approximating the actual distribution of the EM residency programs (81% PGY 1–3 vs 19% PGY1–4), suggests that the sample closely resembles that of the population.^8^ Given our response rate of 65%, it is possible non-response bias affected our results, with participants with less interest or familiarity with telesimulation being less likely to respond. However, the results of our successive wave analysis failed to detect non-response bias for the selected survey questions.

There may be other influences affecting a program’s use of telesimulation that we were not able to capture, and in this survey study we examined only the opinions of faculty and not those of resident learners. Additionally, the literature-based definition of telesimulation we used may be overly broad and encompass more than what typical educators might consider telesimulation. Finally, we acknowledge that the survey was administered in 2022 with in-person learning restrictions just starting to be lifted, and how people are using telesimulation now may be changing. Future work could examine this evolving use of telesimulation within EM residency programs.

## CONCLUSION

This study describes past and planned future use of telesimulation within EM residency programs. A large proportion of EM residencies trialed telesimulation during COVID-19-induced restrictions. Despite relatively low use of telesimulation prior to the pandemic, more EM programs plan to incorporate telesimulation moving forward as a limited portion of their overall simulation curriculum. Opportunities for further innovation and scholarship within this area of simulation education will be possible given this planned continued use.

## Supplementary Information




